# Interactions between 4-thiothymidine and water-soluble cyclodextrins: Evidence for supramolecular structures in aqueous solutions

**DOI:** 10.3762/bjoc.12.54

**Published:** 2016-03-21

**Authors:** Vito Rizzi, Sergio Matera, Paola Semeraro, Paola Fini, Pinalysa Cosma

**Affiliations:** 1 Università degli Studi “Aldo Moro” di Bari, Dipartimento di Chimica Chimica, Via Orabona, 4, 70126 Bari, Italy; 2 Consiglio Nazionale delle Ricerche CNR-IPCF, UOS Bari, Via Orabona, 4, 70126 Bari, Italy

**Keywords:** cyclodextrins, inclusion complex, photodynamic therapy

## Abstract

Since several years the inclusion of organic compounds (guests) within the hydrophobic cavity (host) of cyclodextrins (CDs) has been the subject of many investigations. Interestingly, the formation of inclusion complexes could affect the properties of the guest molecules and, for example, the influence of the delivery system can be a method to improve/change the photochemical behavior of the guest. In particular, very recent studies have shown the protective role of CDs preventing the degradation of the encapsulated guest. Starting from this consideration, in this work, only the structure and complexation mode of the inclusion complexes involving 4-thiothymidine (S^4^TdR, a known photosensitizer) and five CDs, namely 2-hydroxypropyl-α-cyclodextrin (2-HP-α-CD), 2-hydroxypropyl-β-cyclodextrin (2-HP-β-CD), 2-hydroxypropyl-γ-cyclodextrin (2-HP-γ-CD), heptakis-(2,6-di-*O*-methyl)-β-cyclodextrin (DIMEB CD) and heptakis-(2,3,6-tri-*O*-methyl)-β-cyclodextrin (TRIMEB CD) were investigated by different spectroscopic techniques (UV–vis, FTIR–ATR, ^1^H NMR) and cyclic voltammetry analysis (CV). This work is necessary for a prospective research on the photoreactivity of S^4^TdR in aqueous environment and in the presence of CDs to prevent its degradation under irradiation. UV–vis, FTIR–ATR and CV measurements suggested the formation of supramolecular structures involving the employed CDs and mainly the pyrimidine ring of S^4^TdR. ^1^H NMR analyses confirmed such indication, unveiling the presence of inclusion complexes. The strongest and deepest interactions were suggested when TRIMEB and DIMEB CDs were studied. The S^4^TdR affinity towards CDs was also evaluated by using the Benesi–Hildebrand (B–H) equation at 25 °C employing CV and ^1^H NMR methods. The stoichiometry of the interaction was also inferred and it appears to be 1:1 for all examined CDs.

## Introduction

Since several years, supramolecular chemistry has been considered the chemistry of the intermolecular bonds inducing the association of several chemical species with the formation of superstructures [1–3]. The Nobel laureate J. M. Lehn said that “supramolecular chemistry may be defined as chemistry beyond the molecule, bearing on the organized entities of higher complexity that result from the association of two or more chemical species held together by intermolecular forces. Its development requires the use of all resources of molecular chemistry combined with the designed manipulation of non-covalent interactions so as to form supramolecular entities, supermolecules possessing features as well defined as those of molecules themselves” [1].

Starting from this definition, it is worth mentioning that among different kinds of non-covalent interactions, the host–guest type is extensively discussed in literature covering a large field of applications [4]. For example, cyclodextrins (CDs), among host molecules, are reported to occupy an important place in the field of inclusion phenomena [5–7].

CDs are a family of cyclic oligosaccharides and different members of this family have been used widely for several years. More specifically, CDs are manufactured from starch, a polymer with D-glucopyranoside building blocks with both α-1,4- and α-1,6-glycosidic linkages [8]. The most common CDs are α-, β- and γ-CD, and the differences among them are related to the number of α-D-glucopyranose units. While α-CD, characterized by a small cavity size, is hardly used and γ-CD is expensive, β-CD is extensively used because its cavity size is able to allocate a large number of guest molecules [9]. In fact, in the formation of inclusion complexes [10–12] the inner diameter of the cavity of the CDs and the size of the guest molecule play an important role.

CDs are often described as a toroid having a large and narrow cavity. The wider side is formed by the secondary 2- and 3-hydroxy groups, and the narrower side is primarily formed by the primary 6-hydroxy group [13]. The cavity of the CDs is made of oxygen atoms of the glycosidic ring and hydrogen atoms that give the well-known hydrophobic character of the CDs. In addition, the presence of the mentioned free hydroxy groups on the outside of the CDs cavity results in the hydrophilic character of the molecules [6–7,14].

Different chemically modified CDs are known in literature, which are characterized by a higher water solubility compared with the native CDs. These CDs, i.e., 2-HP-CDs, are widely used [15]. An enhanced solubility, both in water and in organic solvents, was also attributed to DIMEB and TRIMEB CDs. Indeed, the formation of DIMEB CD and TRIMEB CD inclusion complexes are reported, for example, by Yu et al. [16] and Nishijo et al. [17], respectively, indicating that they have potential uses as carriers for different drugs for medical applications [18–19]. In fact, for pharmaceutical applications, the improvement of drug stability and solubility play a key role [19]. For example, CDs emerged in biological applications as suitable delivery systems for water-insoluble photosensitizers (PSs) for light-induced photodynamic therapy (PDT) applications [19]. Some authors of this paper have extensively studied the behavior of several PSs in the presence of CDs [19–20] highlighting the important role of the latter.

Interestingly, among PSs the use of thiobases has emerged as a novel approach for PDT applications in several clinical diseases [12,21]. One of the most commonly used thiobase for PDT applications is S^4^TdR, whose triplet state is generated upon photoexcitation with a quantum yield of one [22–26]. Under these conditions the production of reactive oxygen species (ROS), well-known toxic agents against cancer cells, is observed [27–28]. However, one of the major drawbacks with PSs is that most of them decay under prolonged irradiation, which leads to the potentially incomplete destruction of cancer cells. Not surprisingly, Bekalé et al. [27–28] reported that when a PS is irradiated with light, ROS (namely singlet oxygen, superoxide ions and hydrogen peroxide), generated via the excited state of the PS, destroy the PS itself. Because of this, the effort to preserve the PS is one of main issues. Regarding S^4^TdR, as a result of its photodynamic activity, the thiobase can be destroyed by a degradative ROS-mediated oxidation process [26].

Recently, in our papers [26], we have shown the pH-related features and the photoactivity of S^4^TdR in aqueous solution while simultaneously elucidating the nature of products induced by light-stimulated oxidative stress. Evidence for the generation of thymidine (TdR) as the main product was obtained [26]. The use of a delivery system could be a method to improve the photochemical behavior and to prevent the degradation of the thiobase [27]. In fact, literature suggests that one method to avoid the drug degradation is the complexation with CDs, and, as described in [18], the preparation of simple physical mixtures with CDs was already shown to be effective in terms of protective activity. Starting from these considerations, the main goal of this work is to study the ability of the modified nucleoside S^4^TdR to form inclusion complexes with five CDs: 2-HP-α-CD, 2-HP-β-CD, 2-HP-γ-CD, DIMEB and TRIMEB. This is a preliminary work in order to (i) study, in the next future, its photochemical behavior in the presence of CDs, and to (ii) preserve it from oxidative degradation when irradiated during PDT treatments.

In this work, absorption spectroscopic techniques such as, UV–visible absorption spectroscopy, FTIR–ATR and ^1^H NMR with the help of electrochemical analysis were utilized in order to characterize the formation of inclusion complexes between S^4^TdR and CDs. Among the used techniques, ^1^H NMR spectroscopy gave the most direct evidence for the inclusion of S^4^TdR inside the CD cavity [29–30]. In fact, the H3 and H5 atoms of CDs, which are directed towards the interior of the cavity of the CDs showed a significant upfield shift when strong inclusion interactions are present [31–32].

## Results and Discussion

### UV–vis absorption spectroscopy

**Figure 1:**
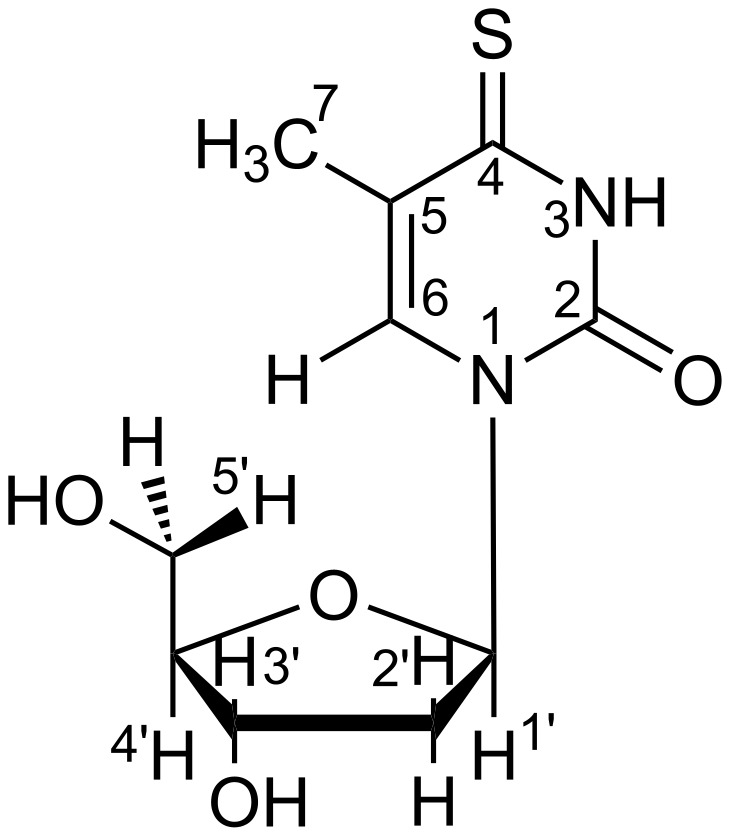
Molecular structure of S^4^TdR. H atoms are also labeled in the Figure.

**Figure 2:**
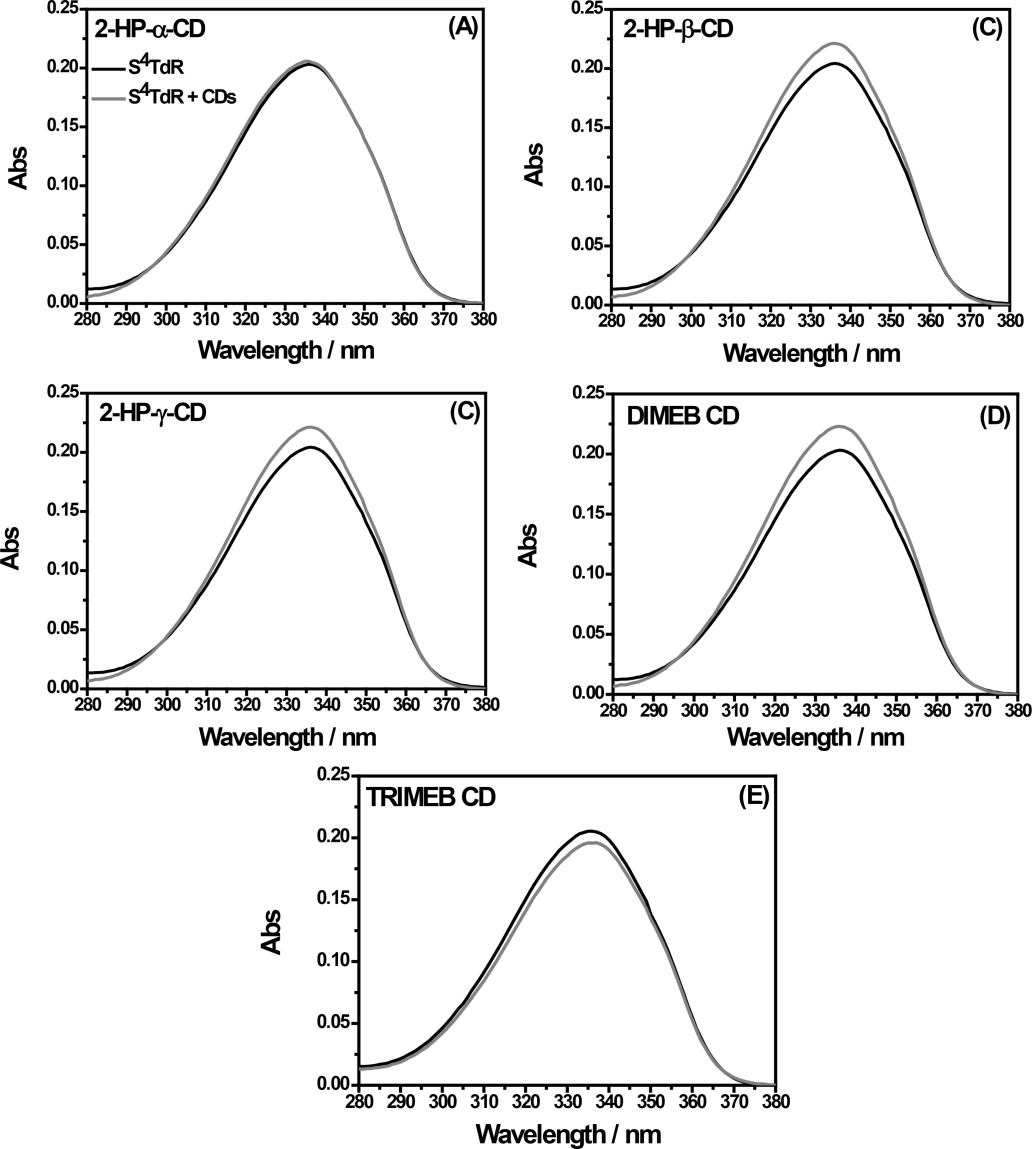
Comparison between UV–vis absorption spectra (detailed view: 280–380 nm) obtained for 1 × 10^−5^ M aqueous solutions of S^4^TdR in absence of CDs (black line), and in presence of CDs (dark gray line), added at a molecular ratio of 1:1. The solution pH is 7.

At first glance, regardless of the structural similarity with thymidine, S^4^TdR (Figure 1) shows a different ultraviolet absorption spectrum. The UV–vis absorption spectrum of 1 × 10^−5^ M aqueous solution of S^4^TdR, was acquired and is reported in Figure 2 (black lines). Subsequently, a comparison in presence of CDs was performed in accordance with similar works reported in literature [32–33] (see dark grey lines in Figure 2).

As reported in Figure 2 (black line), the main absorption band of S^4^TdR shifts from 270 nm (already observed for thymidine) to 337 nm (UVA) [26,34]. The nature of this band was already carefully investigated by some authors of this paper elucidating the behavior of such a nucleoside in aqueous solution. The main form of the nucleoside, absorbing at 337 nm, is the protonated structure reported in Figure 1. The deprotonation, on the nitrogen atom in position 3, started only at pH values greater than 9, with a p*K*_a_ of ca. 9 as described in [26]. Under the latter conditions, the S^4^TdR-main absorption band located at 337 nm in neutral medium shifted to 321 nm. Moreover, the high molar absorption coefficients (ε) evaluated in [26] suggested that the absorption band of S^4^TdR was probably related to a π–π* transition into the second lowest excited singlet (S_2_) state of the molecule. As a result, the delocalized electrons on the pyrimidine ring were supposed to be involved in a transition having a charge transfer character [26]. Taking this into account and with the prospect of an application in biological environments, this preliminary study, related to the possibility of inclusion complexes between S^4^TdR and CDs, was carried out in neutral medium.

Interestingly, as reported in Figure 2, after the addition of CDs, at a molar ratio of 1:1, a significant hyperchromic effect on the main absorption band, located at 337 nm, was observed in the case of 2-HP-β-CD, 2-HP-γ-CD and DIMEB CD (Figure 2b–d). When the TRIMEB CD was used, a hypochromic shift was detected (Figure 2e). While studying 2-HP-α-CD in the presence of S^4^TdR, no significant variations were observed (Figure 2a). In Table 1 the absorbance difference measured at 337 nm in presence and in absence of CDs are summarized.

**Table 1:** Absorbance variation of S^4^TdR when CDs were added at a molar ratio of S^4^TdR/CD = 1:1. ΔAbs_337_ was obtained reading the absorbance value, at 337 nm, of the solution containing S^4^TdR in absence and in presence of CDs.

CD	ΔAbs_337_ (*A*_S4TdR_ − *A*_S4TdR/CD_)

2-HP-α-CD	0
2-HP-β-CD	+0.02
2-HP-γ-CD	+0.02
DIMEB-CD	+0.03
TRIMEB-CD	−0.01

As explained in [31], this change of absorption can be attributed to host–guest type interactions, during which the guest changes its environment from an aqueous medium to the apolar CD cavity inducing the variations observed in the S^4^TdR-absorption spectra. The UV–vis absorption results suggested that the interactions involve mainly the chromophore of S^4^TdR. Besides, when the amount of CDs was further increased, for all examined CDs (with the exception of 2-HP-α-CD, in which not significant changes were measured), both hypochromic and hyperchromic effects of the main absorption band of S^4^TdR were detected. Uekama et al. [35], hypothesized that these observed changes could be due to a perturbation of the electronic energy levels of the guest molecule. Additionally, the micro-environment of the guest changes through a re-organization of solvating water molecules. Undoubtedly, a combination of these two effects could be considered. At this point, since the obtained UV–vis results are not sufficiently clear to definitely prove the formation of S^4^TdR/CDs inclusion complexes, in which the pyrimidine ring is involved, more details were searched through the use of cyclic voltammetry (CV), FTIR–ATR and ^1^H NMR analysis.

### Cyclic voltammetry analysis

**Figure 3:**
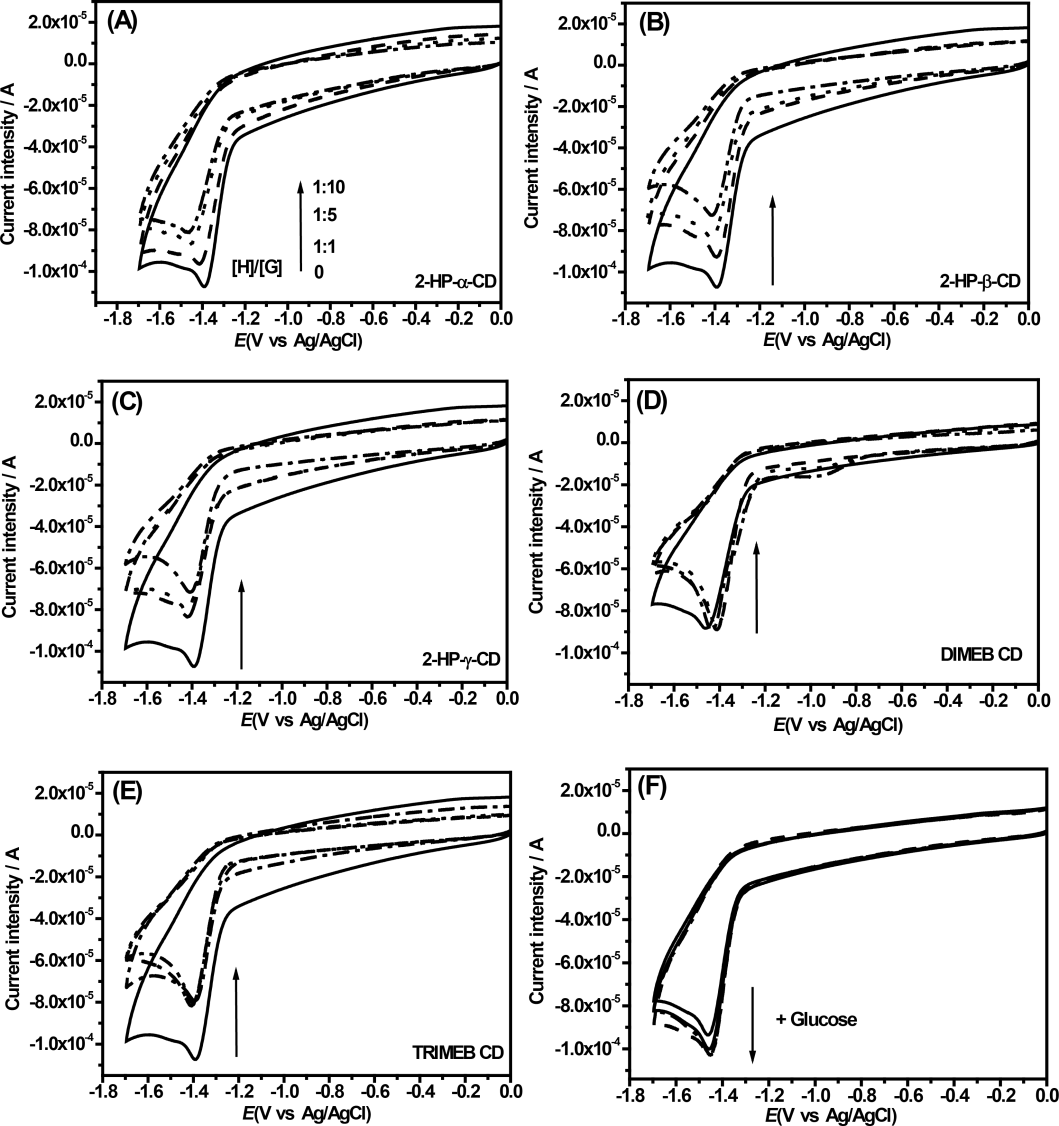
Cyclic voltammetry of aqueous solutions containing S^4^TdR (black solid line) in presence of increasing amounts of CDs, i.e., at molar ratios S^4^TdR/CDs of 1:1, 1:5, 1:10. Supporting electrolyte, LiClO_4_ at 0.1 M. (A) S^4^TdR/2-HP-α-CD, (B) S^4^TdR/2-HP-β-CD, (C) S^4^TdR/2-HP-γ-CD, (D) S^4^TdR/DIMEB CD, (E) S^4^TdR/TRIMEB CD inclusion complexes and (F) S^4^TdR/glucose.

As described in [33], the clear formation of inclusion complexes between S^4^TdR and all employed CDs was investigated by means of electrochemical analyses. In fact, if the employed CDs interact with S^4^TdR, different electrochemical properties would be expected, as described in [36]. The obtained results are reported in Figure 3.

When a S^4^TdR solution is measured with cyclic voltammetry a well-defined irreversible cathodic peak, located at −1.39 V, appeared and was attributed to the reduction of the thiocarbonyl group, (C4=S) (Figure 3**,** solid black line). In particular, as reported in [37] about the electrochemical properties of some thiones, the redox process involving the thionucleoside could be attributable to an electron transfer process centered on the C=S moiety.

**Figure 4:**
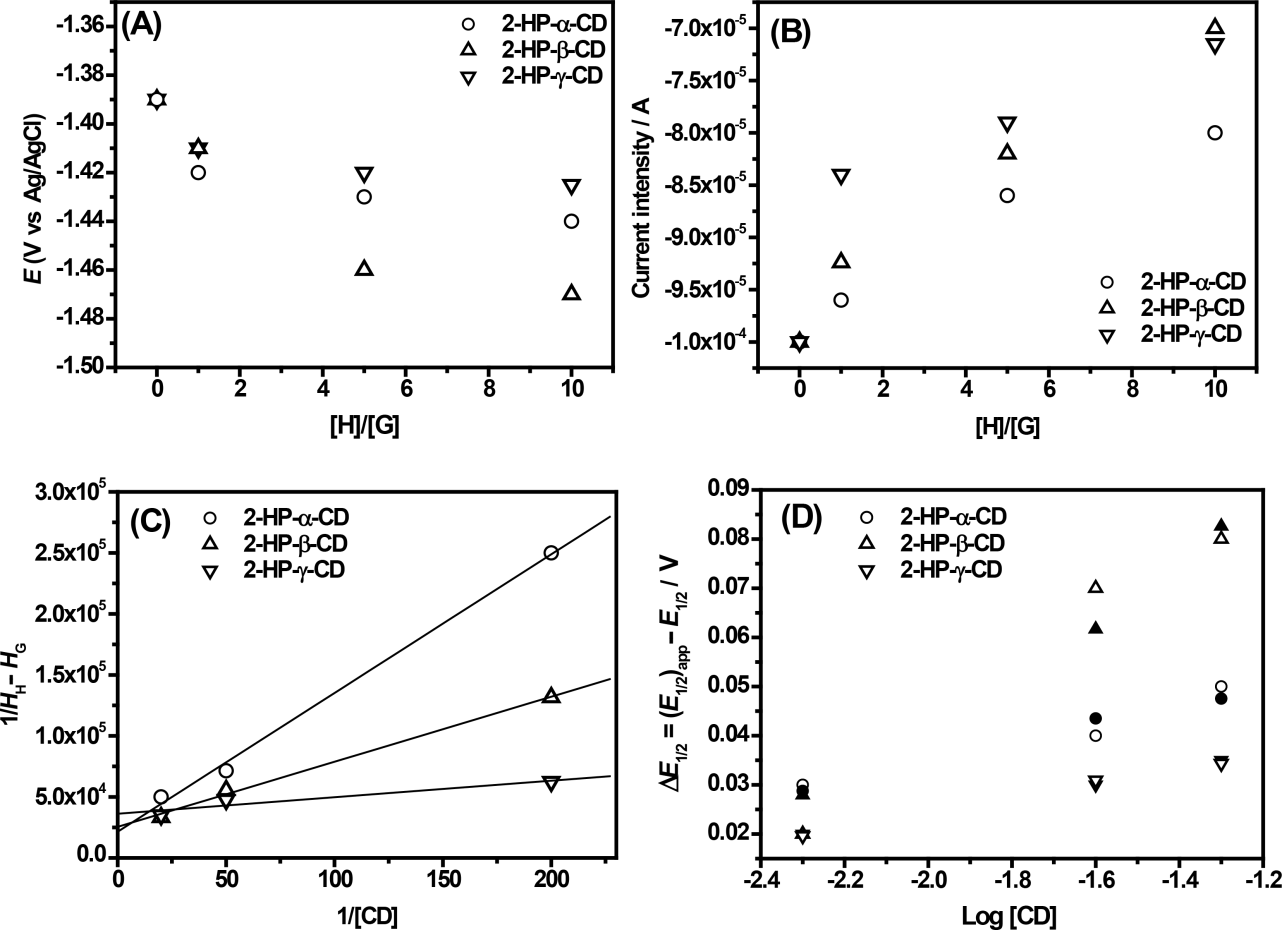
(A) Plot of cathodic potential values and (B) of the corresponding current intensity, obtained for S^4^TdR in absence and presence of 2-HP-CDs, (C) Benesi–Hildebrand plot of 1/*I*_HG_ − *I*_G_ versus 1/[2-HP-CDs] in the presence of S^4^TdR at the initial concentration of 5 × 10^−3^ M, (D) half-wave potential difference of S^4^TdR in the presence of 2-HP-CD ((*E*_1/2_)_CD_) and in the absence of 2-HP-CD (*E*_1/2_) as a function of the total concentration of 2-HP-CD: Experimental values (empty symbols), calculated values (full symbols). Equation 1 (see Experimental part) has been used. The host/guest ratios are 1:1, 5:1 and 10:1.

Interestingly, the addition of CDs in S^4^TdR solutions resulted in remarkable modifications of the electrochemical signals of S^4^TdR (Figure 3A–E). These changes depend significantly on the type of the added CD. The addition of 2-HP-CDs induced a slight negative shift of the cathodic peak potential of S^4^TdR with a concomitant decrease of the current intensity (Figure 3A–C and Figure 4A–B).

After increasing the amount of added CDs, the current intensity (Figure 4B) and the cathodic peak potential (Figure 4A) were largely influenced, indicating as described in [33] that interactions between CDs and S^4^TdR were well-established with the redox center of S^4^TdR located inside the host cavity. The results also indicated, as reported in [38], that S^4^TdR was reduced with more difficulty when it was engaged in the inclusion complexes.

Sabapathy et al. [36] and Semeraro et al. [39] suggested that when host–guest type interactions are established, the diffusion of the inclusion complex from the bulk of the solution to the electrode surface is much slower than that of the guest itself leading at least to the observed current decrease (Figure 3 and Figure 4B) under our experimental conditions. As reported in [36], a decrease of the cathodic peak potential is reasonable because a higher activation energy would be expected under such conditions. As described in [39], this observed increased difficulty is due to the inclusion of the pyrimidine ring inside the cavity of the CDs hindering the interaction with the electrode and reducing the diffusion coefficient of the molecule. The results seem to confirm the variations observed during the UV–vis measurements, in which the pyrimidine ring appeared to be involved in the interactions with CDs.

It is worth mentioning that, as described in [40], our experimental data suggest the presence of a chemical/electrochemical process (CE-type mechanism) where the reduction process is limited by the dissociation step between CDs and S^4^TdR. So, the electron transfer activation occurs after the dissociation of the electroactive guest from the CDs, because S^4^TdR inside the CDs cannot undergo an electrochemical reaction. Then, as described in [40–41], the dissociation of the complex is the preliminary step before the electron transfer process. When inclusion complexes are studied, electron transfer processes are both thermodynamically and kinetically not favored, which in our case confirms the presence of stable inclusion complexes between S^4^TdR and 2-HP-CDs.

When DIMEB and TRIMEB CDs were used a peculiar behavior was observed and the results are reported in Figure 3D,E. When DIMEB CD was added, the S^4^TdR cathodic peak potential shifted slightly towards positive direction with the interesting result that any significant variation was observed furtherly increasing the amount of the CD. Accordingly, the cathodic current intensity was observed to be quite constant. A similar behavior is described in [39] and was explained as the formation of inclusion complexes in which the reduction of the involved groups in the inclusion complex need less activation energy. On the other hand, when TRIMEB CD was considered, while the cathodic peak potential can be considered constant, the current intensity decreases after the first CD addition then it remains constant (Figure 3E). This peculiar scenario, observed for DIMEB and TRIMEB CDs, is described in [2]. Authors explained this behavior with the formation of inclusion complexes with the redox center of the guest molecule located outside the cavity of CD. DIMEB CD and especially TRIMEB CD only offer their hydrophobic domains affecting the electron-transfer processes by changing the diffusion coefficient of the electroactive molecule by inclusion complex formation.

Experiments, in the presence of glucose, mimicking the external interaction between CDs and S^4^TdR, were also performed with the same molar ratios of 1:1, 1:5 and 1:10 and are reported in Figure 3F. Under these conditions the cathodic peak of S^4^TdR appeared unaffected by the addition of glucose, with the cathodic current intensity slightly amplified by external interactions. A contrary behavior than the one observed in the presence of CDs was thus observed, with an almost catalytic effect of glucose. It is noteworthy that the same experiments performed adding glucose at ratios S^4^TdR/glucose of 1:1, 1:5, and 1:10 did not show any influence on the UV–vis spectra of S^4^TdR aqueous solutions (data not shown).

In conclusion the synergic use of UV–vis measurements and CV analysis suggested the presence of inclusion complexes with all examined CDs in which S^4^TdR exhibited different affinity towards them. Interestingly, from CV analysis also the 2-HP-α-CD appeared to form inclusion complexes with S^4^TdR. As a result, for this study, UV–vis techniques appear to be less powerful tools to clearly establish the presence of supramolecular structures involving S^4^TdR and CDs. Furthermore, assuming the formation of a 1:1 host–guest complex, in order to give details about the inclusion complexes the binding constant (*K*) and the stoichiometry of interaction, the Benesi–Hildebrand (B–H) relation (Equation 2, see details in the Experimental section), were determined. In order to determine these constants for the inclusion complexes, the concentration of S^4^TdR was held constant while the concentrations of CDs were varied (see Figure 3). It is worth nothing that the B–H relation was applied only for 2-HP-CDs using the current intensity of the cathodic peak, because of the lack of significant current intensity variations observed for TRIMEB and DIMEB CDs. Figure 4C shows the obtained results. Good linear relationships were obtained from the reciprocal plot of 1/*I*_HG_ − *I*_H_ versus 1/[CD] based on the Equation 2 (r^2^ = 0.998 for 2-HP-α-CD and 2-HP-β-CD; r^2^ = 0.960 for 2-HP-γ-CD). These measurements confirmed the hypothesis of inclusion complexes having a stoichiometry, H/G, of 1:1 [13].

The following *K* values were obtained: (5.0 ± 0.2) × 10^3^ M^−1^, (1.5 ± 0.5) × 10^4^ M^−1^, and (3.6 ± 0.1) × 10^4^ M^−1^, for 2-HP-α-CD, 2-HP-β-CD and 2-HP-γ-CD, respectively, proving the affinity of S^4^TdR to 2-HP-CDs. Unfortunately, because of the experimental results, the *K* values related to DIMEB and TRIMEB CDs were not determined through CV experiments (see NMR section for more details).

In order to get further insight into the complexation constants, the thermodynamic stability of the 2-HP-CD complexes was studied as described in [40], using Equation 1 and the obtained results are reported in Figure 4D. Interesting results were obtained (Figure 4D) by comparing experimental data with theoretical data using the following *K*′ values (referred to the interaction between CDs and the oxidized form of S^4^TdR): *K*′ = (2.0 ± 0.2) × 10^3^ M^−1^ for 2-HP-α-CD, (1.1 ± 0.3) × 10^3^ M^−1^ for 2-HP-β-CD and (1.2 ± 0.2) × 10^3^ M^−1^ for 2-HP-γ-CD. In all examined cases the *K*_0_ values (referred to the interaction between CDs and the reduced form of S^4^TdR) were below 300 M^−1^ suggesting that the reduced form of S^4^TdR is included less efficiently than the oxidized form. The obtained results, considering the magnitude of the obtained *K*′ values, are in agreement with those obtained with the B–H equation confirming a stable interaction between S^4^TdR and CDs. After the assessment of the presence of these supramolecular structures involving CDs and S^4^TdR and in addition to the stoichiometry of the interaction, detailed information was obtained using FTIR–ATR and NMR analysis.

### FTIR–ATR measurements

**Figure 5:**
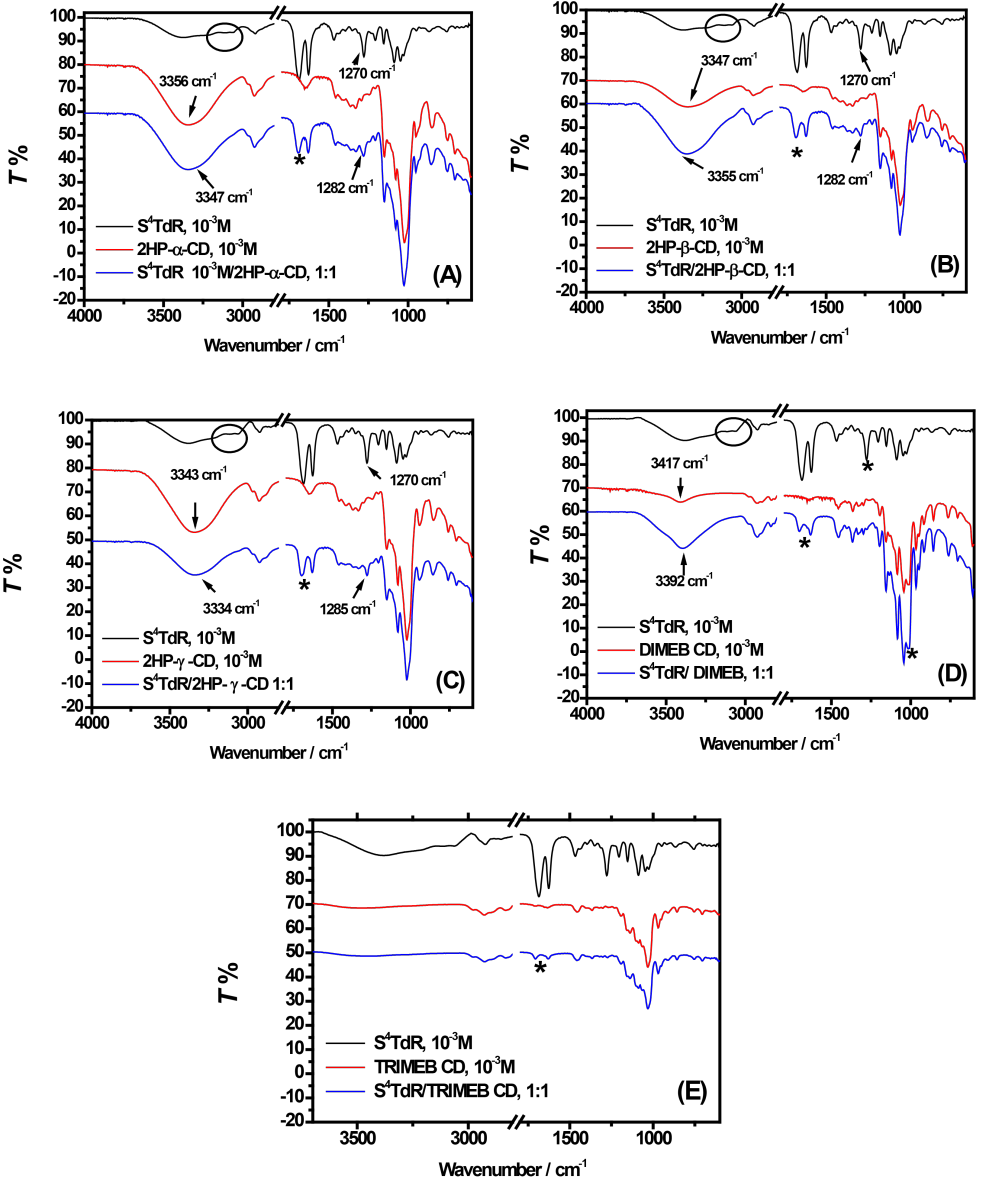
Comparison between detailed views (frequency range: 4000–600 cm^−1^) of the FTIR–ATR spectra obtained for free S^4^TdR (black lines), free CDs (red lines) and their inclusion complexes (blue lines). (A) 2-HP-α-CD/S^4^TdR, (B) 2-HP-β-CD/S^4^TdR, (C) 2-HP-γ-CD/ S^4^TdR, (D) DIMEB CD/S^4^TdR, (E) TRIMEB CD/S^4^TdR inclusion complexes. Wavenumbers between 1800 and 2800 cm^−1^ are omitted because of the absence of IR bands in this region.

FTIR–ATR measurements were performed on S^4^TdR/CDs mixtures in order to confirm the nature of interactions involved in the formation of inclusion complexes, clearly indicated by UV–vis data and successively emphasized by CV measurements. In a preliminary step of the FTIR–ATR study, spectra were acquired on the powders of S^4^TdR and “free CDs” and are reported in Figure 5 (black and red lines). In both cases, the spectra reported in Figure 5 were in excellent agreement with the literature [26,34–35,42–43].

The FTIR–ATR features of S^4^TdR were already carefully investigated in our previous work showing the main characteristic IR bands of such nucleosides (black lines in Figure 5): at 1690 and 1625 cm^−1^ (C2=O and C5=C6 bonds stretching of the S^4^TdR ring), at 1500–1600 cm^−1^ (C=N stretching), at 1465 cm^−1^ (bending of CH_2_ groups), at 1270 cm^−1^ (twisting modes of C4′C5′H_2_OH) and bands at ca. 3350 cm^−1^ (OH groups), at 2900–3000 cm^−1^ (C–H stretching of methyl group), and below 1300 cm^−1^ (deoxyribose ring) [26,34].

The red lines in Figure 5 show the typical spectrum of CDs, already carefully described in literature [42–43]. In brief, typical bands at ca. 3300 cm^−1^ (symmetric and anti-symmetric O–H stretching modes), at 2930 cm^−1^ (symmetric and asymmetric CH stretching) and in the region 1076–1022 cm^−1^ (C–O–C stretching or C–O bonds in the hydroxy groups of HP-CDs) were identified under our experimental conditions. Not surprisingly, the band at 3300 cm^−1^ was not observed when TRIMEB CD was analyzed with FTIR ATR (Figure 5E) and appeared only weakly in DIMEB CD (Figure 5D). This is due to the methylation of the OH moieties. As described in [42], weak absorption bands were detected also in the region of 1100–650 cm^−1^ and can be related to vibrations of the С–Н bonds and to the glucopyranose rind. Further, in the region of 1400–1150 cm^−1^, the absorption bands related to C–H bonds, present in the hydroxy groups of СDs, occurred at 1152 cm^−1^ and at 1332 cm^−1^ [44].

Spectra of the inclusion complexes at stoichiometric ratio of 1:1 looked very similar to those of pure CDs as already reported in [9] and [45]. However, variations involving OH vibrations were observed if when 2-HP-CDs and DIMEB CD were considered (Figure 5). Furthermore, also the typical frequencies of S^4^TdR are largely influenced when the inclusion complexes were formed. In particular, in the presence of all examined CDs, the C=O and C=C vibration modes of the nucleoside (indicated by an asterisk in Figure 5, in the wavenumber region of 1700–1600 cm^−1^) changed distinctly. The dip between the two signals is reduced with important variations of the C=O vibration mode. The effect is even stronger with TRIMEB and DIMEB CDs (Figure 5D,E).

Moreover, when inclusion complexes with 2-HP-CDs were considered (Figure 5B and C), the C=O and C=C signals of S^4^TdR shifted slightly toward higher wavenumbers. When DIMEB and TRIMEB CDs inclusion complexes were measured with FTIR–ATR, the same signals exhibit a largely reduced intensity suggesting the hypothesis of strong interactions between S^4^TdR and these CDs. Additionally, from Figure 5D arises that in presence of DIMEB CD, above signals reversed their intensity highlighting the presence of interactions different from those observed in the presence of TRIMEB CD. Moreover, a significant shift of both C=O and C=C vibration modes was observed from 1690 cm^−1^ to 1711 cm^−1^ and from 1625 cm^−1^ to 1635 cm^−1^, respectively when DIMEB CD was considered. Interestingly, it is known from the literature [46] that upon the formation of inclusion complexes a break of hydrogen bonds occurs with a shift of IR signals to higher wavenumbers. These results were further confirmed by other examples reported in literature, such as the effect on the indomethacin-related carbonyl band and the significant reduction of intensity of similar bands for naproxen in the presence of β-CD observed in [34].

More information was searched looking at the OH vibration modes of CDs when inclusion complexes occurred. As reported in Figure 5 in all examined CDs/S^4^TdR complexes (except for TRIMEB CD, where OH moieties are absent), the stretching of the OH moieties in CDs was shifted towards lower or higher wavelengths than the ones observed in absence of S^4^TdR. Interestingly, Crupi et al. [47] reported that the variations observed under our experimental conditions could be attributed to the presence of novel interactions involving water molecules and CDs [47–48].

Furthermore, during the complexation process S^4^TdR enters into the cavity of the CDs, perturbing the previously existing bonds of the OH groups of CDs. In fact, when a CD and a guest molecule interact with each other, the electron cloud of the guest is perturbed leading to the observed increasing of the wavenumbers [46]. On the other hand, during the host–guest interaction processes, also a decrease in wavenumbers could be observed, in this case the formation of hydrogen bonds and/or van der Waals forces between CDs and the guest molecules can be supposed [49–50].

In all examined CDs, the vibration band at 1270 cm^−1^ related to twisting modes of C4′C5′H_2_OH of S^4^TdR, shifted towards high wavenumbers and disappeared completely when DIMEB and TRIMEB CDs were considered. It is worth mentioning that in the latter cases all vibration bands of S^4^TdR were silent. These results suggested that also the deoxyribose of S^4^TdR partially interacts with the CDs especially when 2-HP-γ-CD or methylated CDs were used (see Figure 5C–E). Indeed in these latter cases strong alterations of the bands were observed (Figure 5). In the presence of CDs, the CH_3_ stretching of the nucleoside (see black circles in Figure 5) was also not detectable under all conditions, suggesting the involvement of this group in the interaction with the host molecules.

The finger print vibration modes of 2-HP-CDs and TRIMEB-CDs appeared not to be significantly influenced through the presence of S^4^TdR, whereas the vibrations in the region of 1000–1100 cm^−1^ of DIMEB CDs, related to the glucopyranose ring (C–O–C stretching), were affected by the presence of the guest (Figure 5D). As reported in [43], the observed variation in the intensity of C–O–C stretching vibration band in the FTIR–ATR spectrum (see asterisk in Figure 5D at ca. 1000 cm^−1^) is attributed to a decrease in the number of hydrogen bonds when the guest compound replaces water molecules inside the CD cavity, explaining also the variations observed at ca. 3400 cm^−1^ [42]. Passos et al. [50], ascribe this behavior to the formation of an inclusion complex .

The host–guest type interactions were further confirmed, analyzing the physical mixture of S^4^TdR and CDs powders (data not shown) [9]. In fact, FTIR spectra of these mixtures showed the typical signals of CDs and S^4^TdR, as a simple superposition of host and guest molecules related bands.

In conclusion, FTIR–ATR measurements better indicate the type of host-guest interactions between CDs and S^4^TdR, with clear differences between 2-HP-CDs and TRIMEB and especially with DIMEB CDs. The differences between 2-HP-CDs and methylated CDs, already evidenced by CV experiments, were thus further confirmed by means of FTIR–ATR analysis. In order to obtain more detailed information, ^1^H NMR experiments were performed obtaining more details about the nature of interactions between S^4^TdR and all examined CDs.

### ^1^H NMR measurements

As described in [51], the insertion of a guest molecule into the hydrophobic cavity of the CDs results in the shift of guest and/or host molecular signals in ^1^H NMR spectra. More specifically, if a guest molecule is located inside CDs cavity, the CDs protons, i.e., H3 and H5 are sensitive to the changed environment [52]. If an upfield shift for H3 and H5 protons is observed, an anisotropic shielding effect arisen from the host–guest association can be considered [53]. Indeed, when the inclusion complex is formed, a large upfield shift of the H3 and H5 protons of the CD, inside the cavity, is usually observed. This behavior can be better explained when the guest molecule has an aromatic ring. In fact, the effect is attributed to the ring current of the aromatic guest located inside the cavity of the CD [54]. In particular, the magnetic anisotropy effect can be taken in consideration to explain this behavior [55]. But also when an aliphatic moiety enters the hydrophobic cavity, an influence on H3 and H5 protons can be expected as reported in [56].

After this assessment, for the sake of comparison with other techniques and in order to give more detailed information, as a preliminary step of the study based on ^1^H NMR spectroscopy, spectra were acquired both for free S^4^TdR and CDs and for their mixture. Results were in excellent agreement with literature. Indeed, the typical NMR spectra of S^4^TdR and CDs were obtained as described in [26,52,57].

Concerning S^4^TdR details can be found in our previous work [26]. In particular, a signal for the 6-H proton (see the numbering used in Figure 1) was observed at 7.77 ppm. A signal of the 7-CH_3_ protons was observed at 2.10 ppm. The deoxyribose protons were all found in the range of 2.3–6.3 ppm. When 2-HP-α-CD/S^4^TdR mixtures were considered, both H3 and H5 protons of the CD appeared unaffected by the presence of the guest and variations were observed only when the H/G ratio was set to 10, observing a slight upfield shift only for proton H3 (Δδ = −0.0036, see Equation 3). As described in [9], the other 2-HP-α-CD protons located outside the cavity showed negligible changes in their chemical shifts upon complexation if compared to the shift obtained for the H3 proton.

Interesting results were obtained when the protons of the guest molecule were considered. The chemical shifts of the guest protons are often reported as an indication of the inclusion complex formation [10]. As reported in Figure S1A (Supporting Information File 1), in which the observed Δδ values (see Equation 3) are shown, the most part of S^4^TdR protons exhibited an oscillating behavior, when the amount of 2-HP-α-CD was increased. More specifically, these protons were shifted slightly upfield and this behavior is attributed to the presence of electronegative groups [55]. In other words these protons experienced a more polar surrounding than the ones observed in absence of CD, suggesting that S^4^TdR was partially located near the cavity of 2-HP-α-CD. As reported in [57] an upfield shift of protons indicates that these protons are close to host atoms rich of π-electrons [58], which in this case were associated with the oxygen atoms of the CDs, but also reflected some conformational changes produced by the inclusion. Moreover, it is known [9], that signals of protons located outside the cavity of CDs exhibit an upfield shift, attributable to the C–C bond anisotropy effect.

Interesting results were obtained when the 7-CH_3_ protons of S^4^TdR were considered. In fact, as shown by the empty black circles in Figure S1A (Supporting Information File 1), Δδ_H7_ followed a linear trend further downfield when the amount of CD was increased. As described in [10], the inclusion complex occurred under our conditions involving mainly the methyl group of the S^4^TdR. Indeed, the observed downfield shift in Figure S1A (Supporting Information File 1) is related to changes in the polarity due to the inclusion of the S^4^TdR into the cavity of the CDs [59]. The results, related to the encapsulation of CH_3_ group, were emphasized considering the observation reported in [56]: when aliphatic protons enter the hydrophobic cavity of CD, no variations of the signals of H3 and H5 protons are expected. Additionally information arises from the observed non-linear trend followed by the 6-H protons, when the amount of 2-HP-α-CD was increased. This behavior indicated the slight tendency of 2-HP-α-CD to interact partially with the 6-H proton directly linked to the pyrimidine ring. In conclusion, when 2-HP-α-CD was considered the observed lack of variation of the signals of H3 and H5 protons, with the trend followed by S^4^TdR protons suggested that the pyrimidine ring of the guest molecule was only partially entrapped inside the CD cavity and that the aliphatic 7-CH_3_ protons, which are not able to significantly shift the protons located in the inner cavity of CD, were involved in this interaction [56]. These results are better explained considering also FTIR–ATR and CV data. In fact, the former suggested the involvement of the methyl and the C=O group in the interaction with CD and a partial involvement of the deoxyribose ring with only the CH_2_OH moiety, the latter showed the participation of the C=S moiety. So, the formation of host–guest-like supramolecular structures can be hypothesized.

After increasing the inner diameter of the CD, from 5.7 Å for 2-HP-α-CD to 7.8 Å for 2-HP-β-CD [13], the H3 signals of 2-HP-β-CD, showed a significant variation at all examined H/G ratios (Δδ = −0.0036). However, the observed upfield shift of the H3 protons did not follow a linear trend. The H5 protons exhibited variations only at high concentrations of CD (H/G ratio of 10, Δδ = −0.0012). The results indicated that in this case the efficiency of the formation of inclusion complexes was increased by the larger diameter of 2-HP-β-CD.

These data were further corroborated by considering the S^4^TdR protons (see Figure S1B, Supporting Information File 1). Indeed, it was clear that only 7-CH_3_, and 5′a-H and 5′b-H, and to a lesser degree 6-H protons, were affected by the presence of CDs, following a trend with increasing CD concentrations. Moreover, for these protons, a downfield shift was observed. On the other hand, the clear 1′-H upfield shift, observed in this case, suggested (like before for 2-HP-α-CD ) that the deoxyribose ring of S^4^TdR was located outside the cavity of the CD experiencing the polar medium of OH moieties as already suggested in [55]. Accordingly, the supposed interactions involved when 2HP-α-CD was analyzed, were further confirmed when 2-HP-β-CD was considered. Also in this case, the results were in excellent agreement with FTIR–ATR and CV measurements.

When the largest CD 2-HP-γ-CD was used, all the guest protons showed a downfield shift (Figure S1C, Supporting Information File 1), while the H3 and H5 protons of the CD remained unchanged. This indicates that the largest CD cavity (9.5 Å) [13] encapsulated the ribose ring, too. Interestingly, when the amount of 2-HP-γ-CD was increased, the S^4^TdR protons 2′-H and 5′-H appeared upfield-shifted (Figure S1C, Supporting Information File 1). In other words, at a S^4^TdR/CD ratio of 1:10, the latter protons experienced the polar environment near the cavity of 2-HP-γ-CD. The cathodic potential shift observed in the CV analysis suggested that the inclusion complex is tilted, involving the –CH_3_ and C=S moieties of the guest together with a part of the deoxyribose ring. Since the signals of the H3 and H5 protons of the CD did not change, the effect of the complete entrapment of the S^4^TdR aromatic ring cannot be considered.

**Figure 6:**
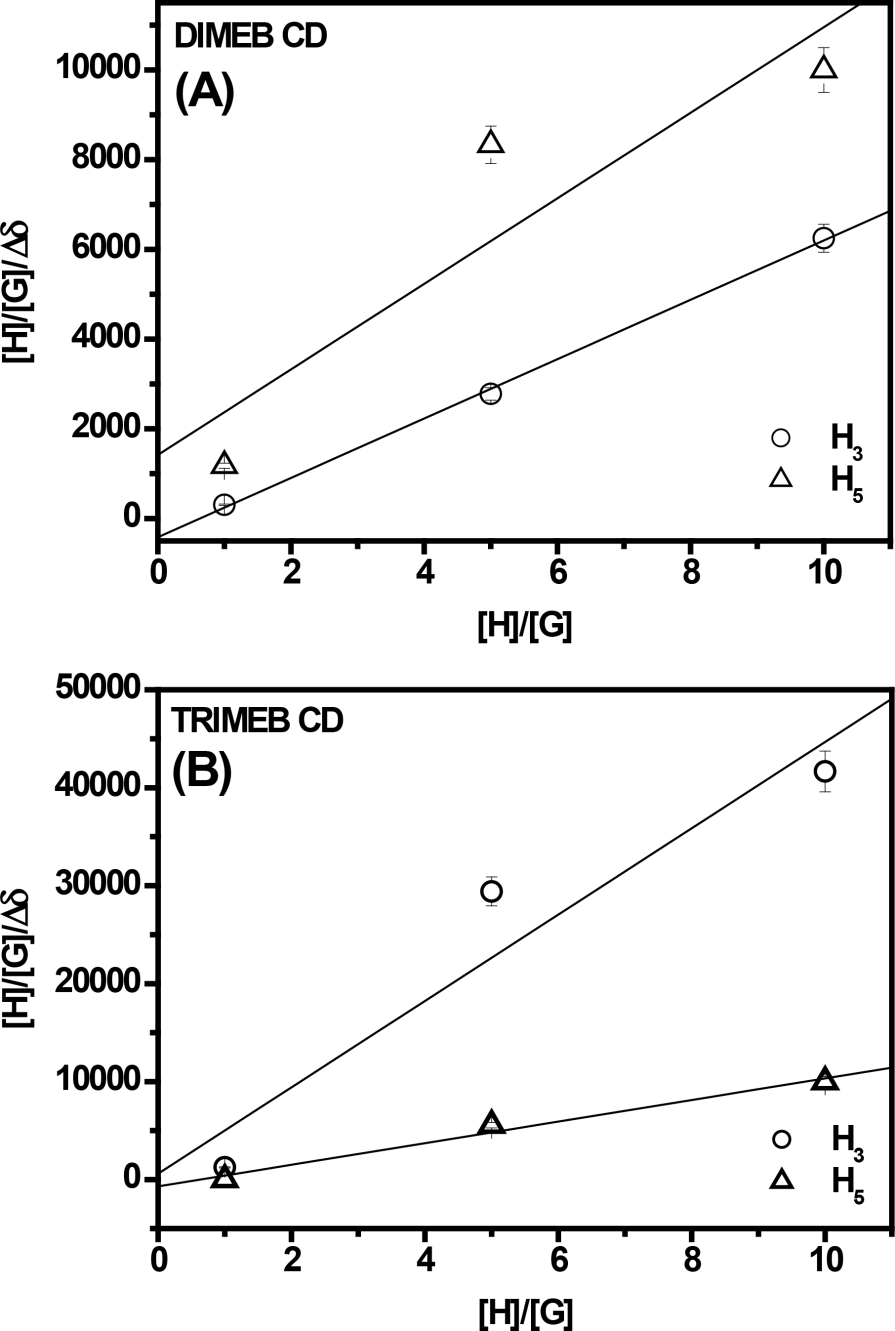
Modified Benesi–Hildebrand plot of [H]/[G]/Δδ versus [H]/[G] in the presence of S^4^TdR at an initial concentration of 1 × 10^−3^ M. The reported host/guest ratios are 1:1, 1:5 and 1:10. Δδ represents the difference between the observed chemical shift of H3 and H5 protons of CDs, in absence and presence of the guest molecule, i.e., Δδ = δ_(complexed state)_ − δ_(free state)_. (A) DIMEB CD/S^4^TdR, (B) TRIMEB CD/S^4^TdR inclusion complexes.

In accordance with FTIR–ATR and CV analysis, remarkable results were obtained when DIMEB and TRIMEB CDs were used. Both H3 and H5 protons of CDs were affected by the presence of S^4^TdR and were shifted upfield. In this case the Δδ values for both protons were calculated (see Figure 6A and Figure 6B, which will be discussed later in the text). In particular the H3 and H5 protons of TRIMEB CD showed variations only at H/G ratios greater than 1. Rekharsky et al. [60] discuss these variations, namely Δδ_H5_ and Δδ_H3_, and their relative ratios, Δδ_H5_/Δδ_H3_, considering the ratio as an indication of the stability of the complex. If the ratio Δδ_H5_/Δδ_H3_ is below 1, the insertion of the aromatic ring of the guest into the host cavity involves mainly the H3 proton; in other words the inclusion is not strong. On the other hand, the opposite effect is indicative of deepest interactions [60].

The results are shown in Figure 6 in which a modified B–H equation, as suggested by [10], was used both for DIMEB and TRIMEB CDs. In particular, by plotting Δδ_H5_*/*Δδ_H3_ as a function of the ratio [H]/[G], a linear trend was observed supporting the formation of an inclusion complex with a 1:1 stoichiometry, as already observed for 2-HP-CDs.

Moreover, the changes of Δδ_H5_/Δδ_H3_ suggested that the pyrimidine ring of S^4^TdR completely entered into the nanohydrophobic cavity when the methylated CDs were used. The inclusion complex was deeper when DIMEB CD was used then the one observed for TRIMEB CD. In fact, Δδ_H5_/Δδ_H3_ is greater than 1 when DIMEB CD was considered.

These results were corroborated considering the S^4^TdR protons and their chemical shifts. When DIMEB CD/S^4^TdR inclusion complexes were studied (see Figure S1D, Supporting Information File 1), a very important effect was observed when the 6-H proton, directly linked to the double bond of the nucleobase, was considered. The 6-H proton appeared to be significantly downfield shifted, with others protons that slightly followed the same trend with the exception of the proton 1′-H. Indeed, the latter was shifted upfield indicating the increased polarity of the perceived environment [55]. A similar effect appeared when TRIMEB CD was used, too. However, in this case, also protons 2′-H were shifted upfield in the presence of CD and, in addition, the protons 7-CH_3_ of S^4^TdR appeared downfield shifted like the 6-H proton. In conclusion, from the obtained results, in which, in the case of TRIMEB CD, the proton H5 was slightly influenced compared to DIMEB CD, the complex, between TRIMEB CD and S^4^TdR appeared to be weaker than DIMEB CD/S^4^TdR, but deeper than the ones observed in the presence of hydroxypropyl cyclodextrins [60].

The results for methylated CDs were in accordance with FTIR–ATR and CV analysis. In fact, the infrared spectra showed the strongest variation and CV suggested that the C=S group could be outside the cavity of CDs. The differences between the two CDs could be ascribed to the number of methyl groups present on the surface of CDs. TRIMEB CD is completely methylated, while DIMEB CD is methylated only in positions 2 and 6, with an OH moiety in position 3, which is able to form OH-mediated hydrogen bonds, involving the thiol moiety [61]. In other words when DIMEB CD was used, the presence of both CH_3_ and OH moieties induced a tight host–guest interaction in which the C=S group is coordinated outside the cavity through hydroxy groups via H bonds.

## Conclusions

With the prospect to evaluate the photostability of 4-thiothymidine (S^4^TdR) in the presence of CDs, the formation of inclusion complexes involving aqueous solutions of S^4^TdR and five CDs (namely 2-HP-α-CD, 2-HP-β-CD, 2-HP-γ-CD, DIMEB CD, and TRIMEB CD) were investigated by different spectroscopic techniques (UV–vis, FTIR–ATR, ^1^H NMR) and cyclic voltammetry analysis (CV).

The employed methods indicated that S^4^TdR was able to interact with all examined CDs, showing a stronger affinity towards DIMEB and TRIMEB CDs. CV experiments suggested that when 2-HP-CDs were considered, the redox center of the S^4^TdR was certainly involved in the hydrophobic cavity of the host, as evidenced by the shift of the cathodic peak potential of the CDs towards low values with the concomitant decrease of the current intensity. These results indicate that in presence of 2-HP-CDs, a consecutive chemical–electrochemical (CE) mechanism can be assumed suggesting that before any electrochemical processes S^4^TdR is released from 2-HP-CDs.

When TRIMEB and DIMEB CDs were analyzed, a different behavior was observed. The cathodic peak potential of DIMEB CD shifted slightly in a positive direction, at the first addition of CD (without any further variations observed when the amount of CD was increased. When TRIMEB CD was considered the cathodic peak potential and the current intensity remained constant. These results suggested that the C=S moiety of the S^4^TdR could be located outside the cavity of DIMEB and TRIMEB CDs.

FTIR–ATR and ^1^H NMR further corroborated these results. In fact, FTIR–ATR experiments showed significant variations in the IR spectrum of the guest showing that the methyl group of S^4^TdR, in position 7, and the pyrimidine ring, more specifically the C=O and C=C groups, were mainly involved in the formation of the inclusion complexes. Strong interactions, involving the mentioned groups, were largely observed when DIMEB and TRIMEB CDs were considered.

These results were further confirmed by ^1^H NMR, showing, in addition, that different interactions were established between S^4^TdR and CD depending on the type of CD type. In particular, only when the methylated CDs were used, there was a strong variation of the signals of the H3 and H5 protons, located inside the cavity of CDs, indicating a stronger affinity of S^4^TdR towards these CDs. By means of the Benesi–Hildebrand equation applied to the NMR data also the stoichiometry of the formed inclusion complex was elucidated, which appears to be 1:1.

In general, the present study confirms that the interactions between S^4^TdR and the used CDs occurred, and a tight complex was obtained when DIMEB and TRIMEB CDs were used. At this point the influence of a delivery system could be a potential method to improve/change the photochemical behavior of the thiobase. In particular, the protection of S^4^TdR, as photosensitizer, from degradation through reactive oxygen species during photodynamic therapy applications could be attained in the presence of all analyzed CDs. A study in this direction, with a good perspective, is being carried out in our laboratory.

## Experimental

### Chemicals

2-HP-α-CD, 2-HP-β-CD, 2-HP-γ-CD, DIMEB and TRIMEB CDs were purchased from Sigma-Aldrich (Milan, Italy) and used without further purification. The same commercial source was adopted for D_2_O and LiClO_4_ (99.99% trace metals basis). 4-Thio(2’-deoxy)thymidine (S^4^TdR), having 99+% purity was purchased from Carbosynth (Compton, Berkshire, UK). S^4^TdR and CDs stock solutions (at concentrations of 10^−2^ M and 5 × 10^−2^ M, respectively) were prepared in doubly distilled water or, in D_2_O for ^1^H NMR analysis and in the presence of LiClO_4_ (0.1 M) for CV experiments. The solutions were stored at 4 °C.

### Sample preparation

Inclusion complexes were prepared by adding directly different amounts of CDs, at several host/guest (H/G) ratios, in a aqueous solution of S^4^TdR. For ^1^H NMR experiments, the solutions were prepared by dissolving S^4^TdR and CDs in D_2_O and spectra were recorded with DHO as internal standard according to Trapani et al. [32].

The H/G ratio was set to 1:1, 5:1 and 10:1 with S^4^TdR concentrations of 1 × 10^−3^ M for FTIR–ATR and ^1^H NMR experiments and 5 × 10^−3^ M for CV analysis. In the latter, the high amount of S^4^TdR was necessary in order to detect significant signals in measurements. UV–vis measurements were carried out using a S^4^TdR concentration of 1 × 10^−5^ M. Before use, these solutions were maintained under agitation for 10 min at room temperature.

### Analysis

#### UV–vis measurements

UV–vis spectra were recorded in the range of 200–600 nm, at a scan rate of 1 nm/s, using a Varian CARY 5 UV–vis–NIR spectrophotometer (Agilent Technologies Inc., Santa Clara, CA, USA).

#### FTIR–ATR measurements

As described in [26], a FTIR spectrometer 670-IR (Agilent Technologies Inc., Santa Clara, CA, USA), the resolution of which was set to 4 cm^−1^ was used to perform our analysis. Spectra were collected in the range of 600–4000 cm^−1^ range and 32 scans were integrated for each analysis.

#### ^1^H NMR measurements

As described in [26], ^1^H NMR measurements were carried out using a Bruker AVANCE III 700 MHz spectrometer (Bruker BioSpin GmbH, Rheinstetten, Germany). A 5 mm 1H/D-BB probe head, with z-gradient, automated tuning and matching accessory, and an accessory for temperature control (BTO-2000) were used.

#### Cyclic voltammetry measurements

As described in [2], the range between −1.7 and 0 V (rate 0.1 V/s) was chosen to perform our experiments using an Autolab PGSTAT10 potentiostat/galvanostat. Experiments were conducted at room temperature under argon atmosphere using a cell (10 mL) with three electrodes. A glassy carbon electrode (3 mm diameter) was our working electrode, while the reference and counter electrodes were Ag/AgCl, Cl^−^ (saturated KCl) and a platinum wire. In our experiments, the working electrode was polished with alumina and washed with water before each experiment. In detail, the glassy carbon electrode was first rinsed with tap water, followed by a methanol rinse and carefully wiped dry with a fresh lab tissue. Then, the electrode was treated with alumina slurry, rinsed well with distilled water and sonicated for about 3 min in a shallow amount of distilled water to remove residual abrasive particles. After the ultrasound bath treatment, the electrode was rinsed again with distilled water, again briefly with methanol and wiped dry under nitrogen flow. A 0.1 M solution of LiClO_4_ was used as supporting electrolyte. Solutions were analyzed after 30 min, a period in which the system was degassed by the argon flow. All experiments were performed at 298 K.

The binding constants *K* and the stoichiometry of the interaction of S^4^TdR and CDs were determined according to the modified Benesi–Hildebrand (B–H) relation (Equation 2) [9] assuming the formation of host–guest complexes with stoichiometry of 1:1. In Equation 2, *I*_G_ represents the cathodic current intensity of the guest molecule, in absence of CDs, and *I*_HG_ is the cathodic current intensity of the inclusion complex of S^4^TdR and CD. *I*_HG_ − *I*_G_ is the difference between the cathodic current intensity of inclusion complex and S^4^TdR alone. Δ*I* is the difference between the molar peak current coefficient of the inclusion complex and S^4^TdR.

[2]
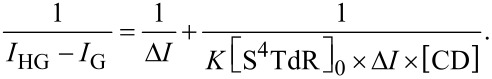


It is worth mentioning that Equation 2 was used only in the case of 2-HP-CDs, the only case in which appreciable variations were observed concerning the electrochemical properties of the guest in the presence of CDs.

As described in [40] the thermodynamic stability of the studied complexes, S^4^TdR and 2-HP-CDs, were evaluated plotting the differences between the half-wave potential (Δ*E*_1/2_) in the presence of CD ((*E*_1/2_)_app_) and the half-wave potential in the absence of CD (*E*_1/2_) versus the concentration of 2-HP-CDs (see Equation 1).

[1]
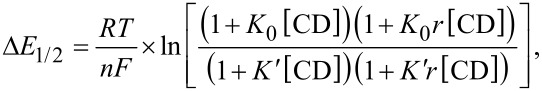


where *K*_0_ and *K*′ represent the inclusion complex constants related to S^4^TdR in its reduced and oxidized form, respectively. The *r* values, in accordance with Buriez et al. [40] are calculated as the ratio of the molecular weight of S^4^TdR and its related inclusion complexes (see [40–41] for more details).

The observed variation of ^1^H NMR chemical shifts of CDs or S^4^TdR protons induced upon complexation were calculated in order to confirm the interaction between CDs and S^4^TdR according to the following equation (Equation 3):

[3]
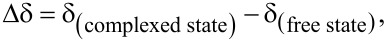


where δ_(complexed state)_ and δ_(free state)_ are the chemical shifts of the CDs/S^4^TdR protons in the complexed state in the free state, respectively. A plot of the variation of the chemical shift (Δδ) obtained for H3 and H5 protons of CDs in the form of ([H]/[G])/Δδ (H3 or H5) as a function of [H]/[G] was also used in order to give information about the complexation. The latter relation represents a modified Benesi–Hildebrand plot adapted for NMR data [31]. It is worth mentioning that this equation was used only in the case of TRIMEB and DIMEB CDs, cases in which significant variations were observed concerning the H5 and H3 protons of the CDs.

## Supporting Information

File 1 Differences between the observed chemical shifts of S^4^TdR protons in presence of the CDs.
