# Supplementary material for: Interactions between 4-thiothymidine and water-soluble cyclodextrins: Evidence for supramolecular structures in aqueous solutions
Source: Beilstein J Org Chem. 2016 Mar 21;12:549–63. doi: 10.3762/bjoc.12.54 (PMC4901997; doi:10.3762/bjoc.12.54)
Supplement: File 1 — Differences between the observed chemical shifts of S4TdR protons in presence of the CDs. [file Beilstein_J_Org_Chem-12-549-s001.pdf]

Supporting Information

for

Interactions between 4-thiothymidine and water-soluble cyclodextrins: Evidence for supramolecular structures in aqueous solutions

Vito Rizzi[1], Sergio Matera[1], Paola Semeraro[1], Paola Fini[2] and Pinalysa Cosma*[§1,2]

Address: [1]Università degli Studi "Aldo Moro" di Bari, Dipartimento di Chimica Chimica, Via Orabona, 4, 70126 Bari, Italy and [2]Consiglio Nazionale delle Ricerche CNR-IPCF, UOS Bari, Via Orabona, 4, 70126 Bari, Italy

Email: Pinalysa Cosma* - pinalysa.cosma@uniba.it

*Corresponding author

[§]Tel. +39 0805443443

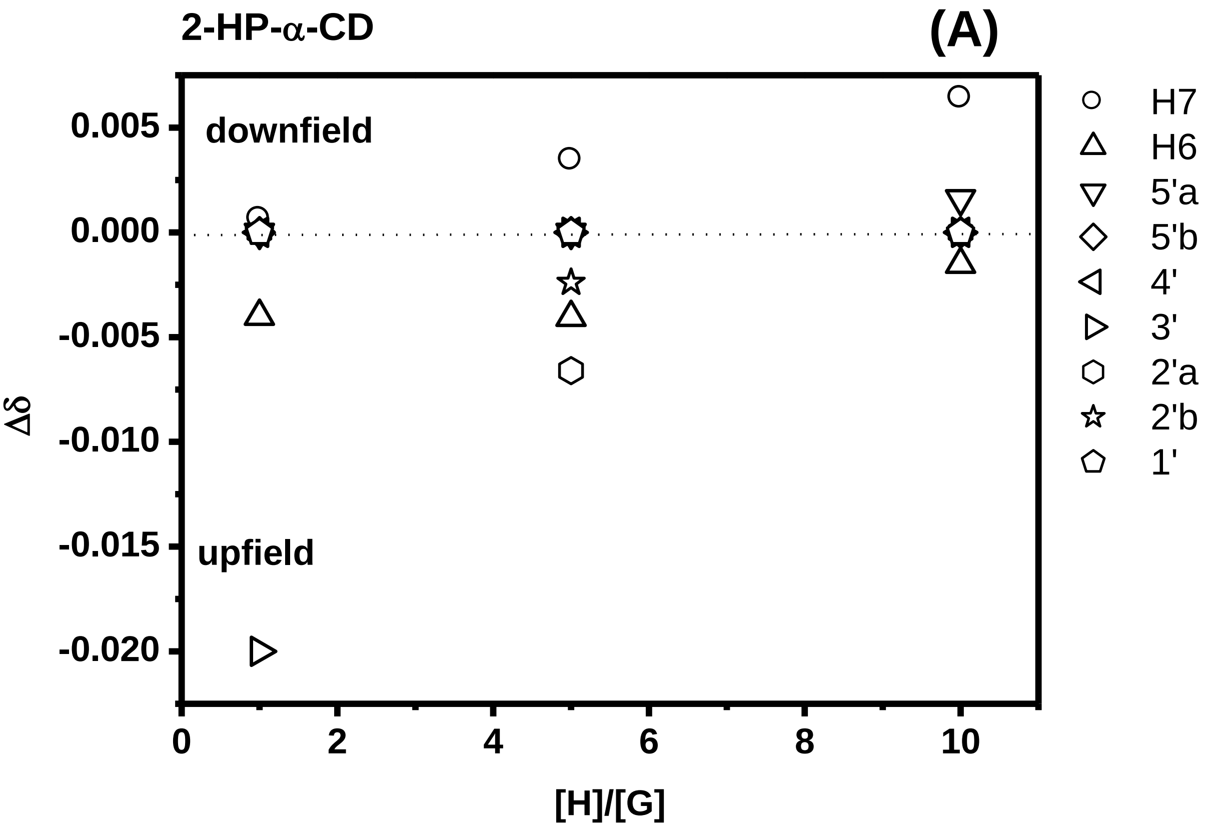

2-HP-α-CD
(A)
downfield
upfield
Δδ
[H]/[G]
0.005
0.000
-0.005
-0.010
-0.015
-0.020
0
2
4
6
8
10
H7
H6
5'a
5'b
4'
3'
2'a
2'b
1'

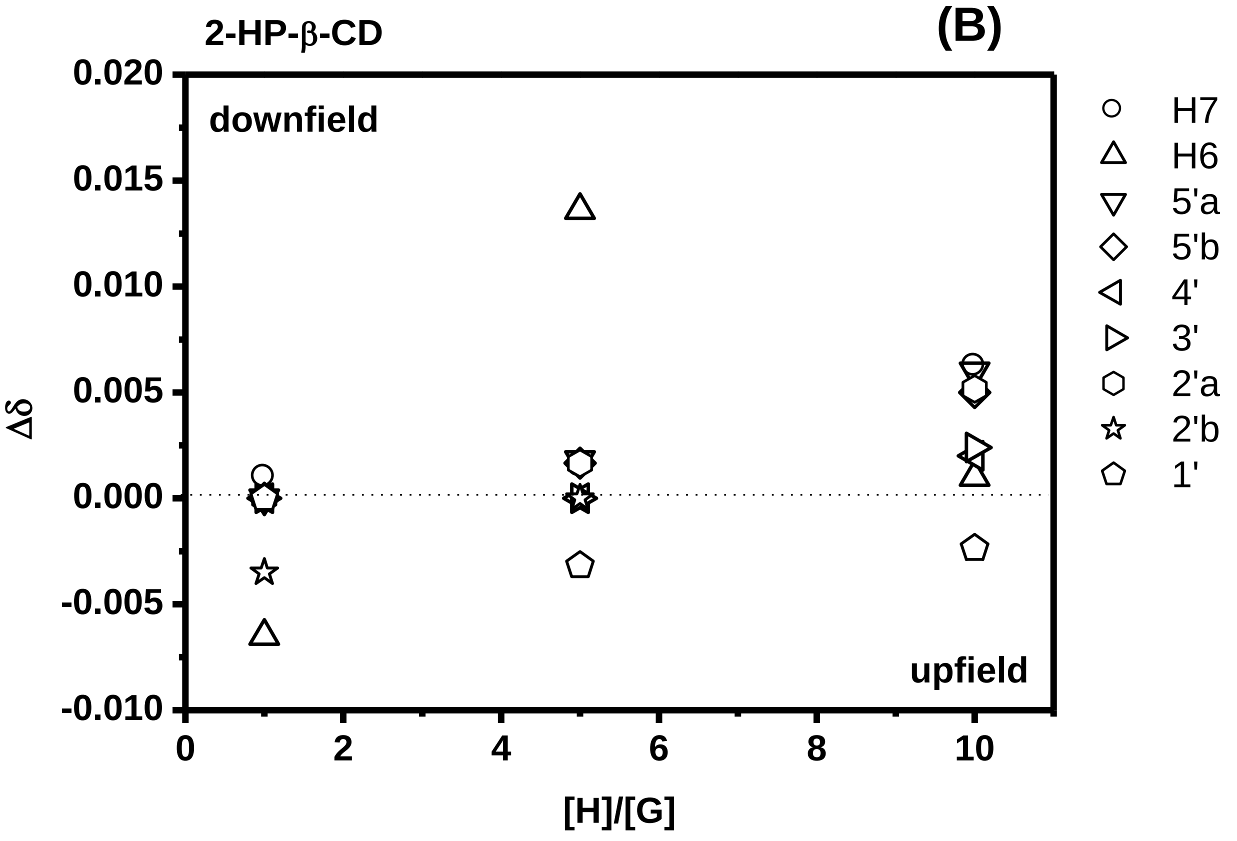

2-HP-β-CD
(B)
downfield
upfield
Δδ
[H]/[G]
0.020
0.015
0.010
0.005
0.000
-0.005
-0.010
0
2
4
6
8
10
H7
H6
5'a
5'b
4'
3'
2'a
2'b
1'

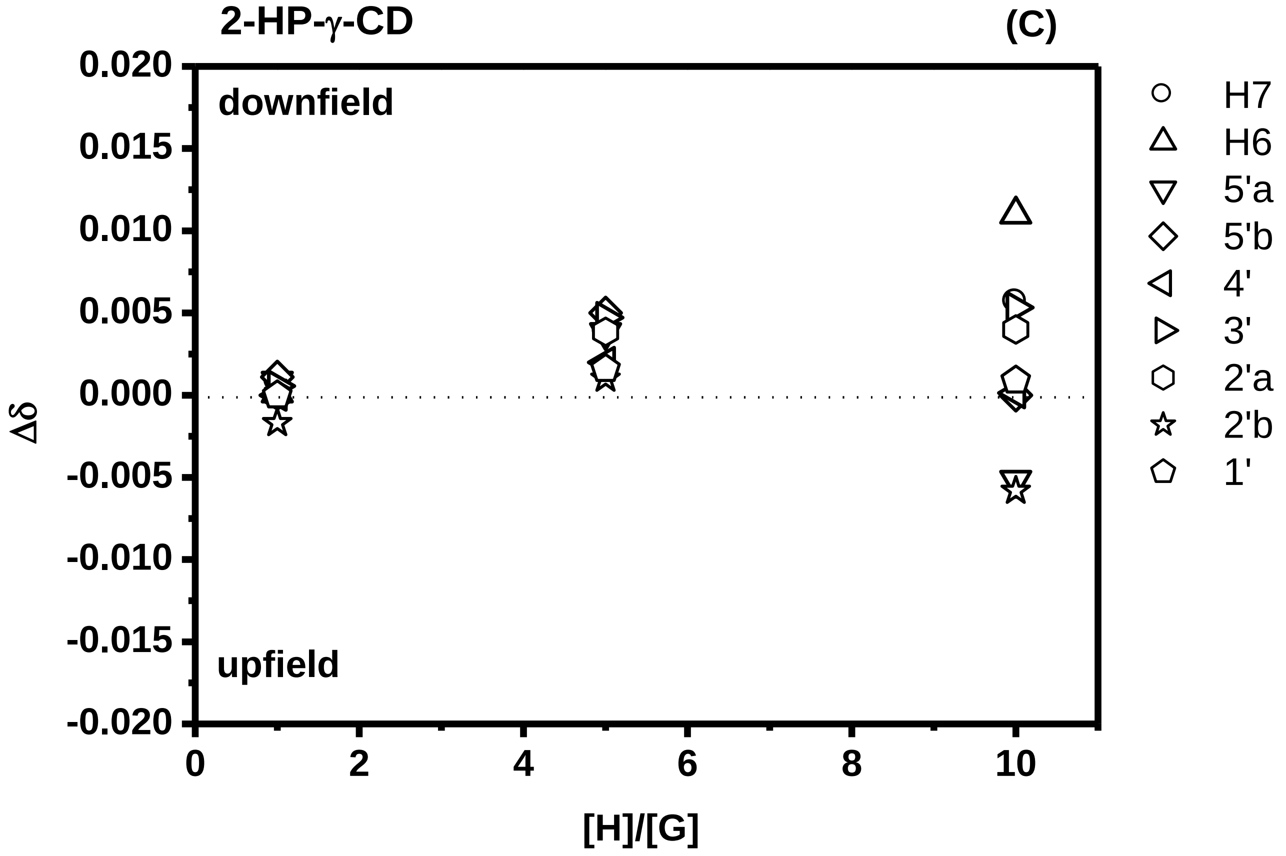
2-HP-γ-CD
(C)
downfield
upfield
Δδ
[H]/[G]
0.020
0.015
0.010
0.005
0.000
-0.005
-0.010
-0.015
-0.020
0
2
4
6
8
10
H7
H6
5'a
5'b
4'
3'
2'a
2'b
1'

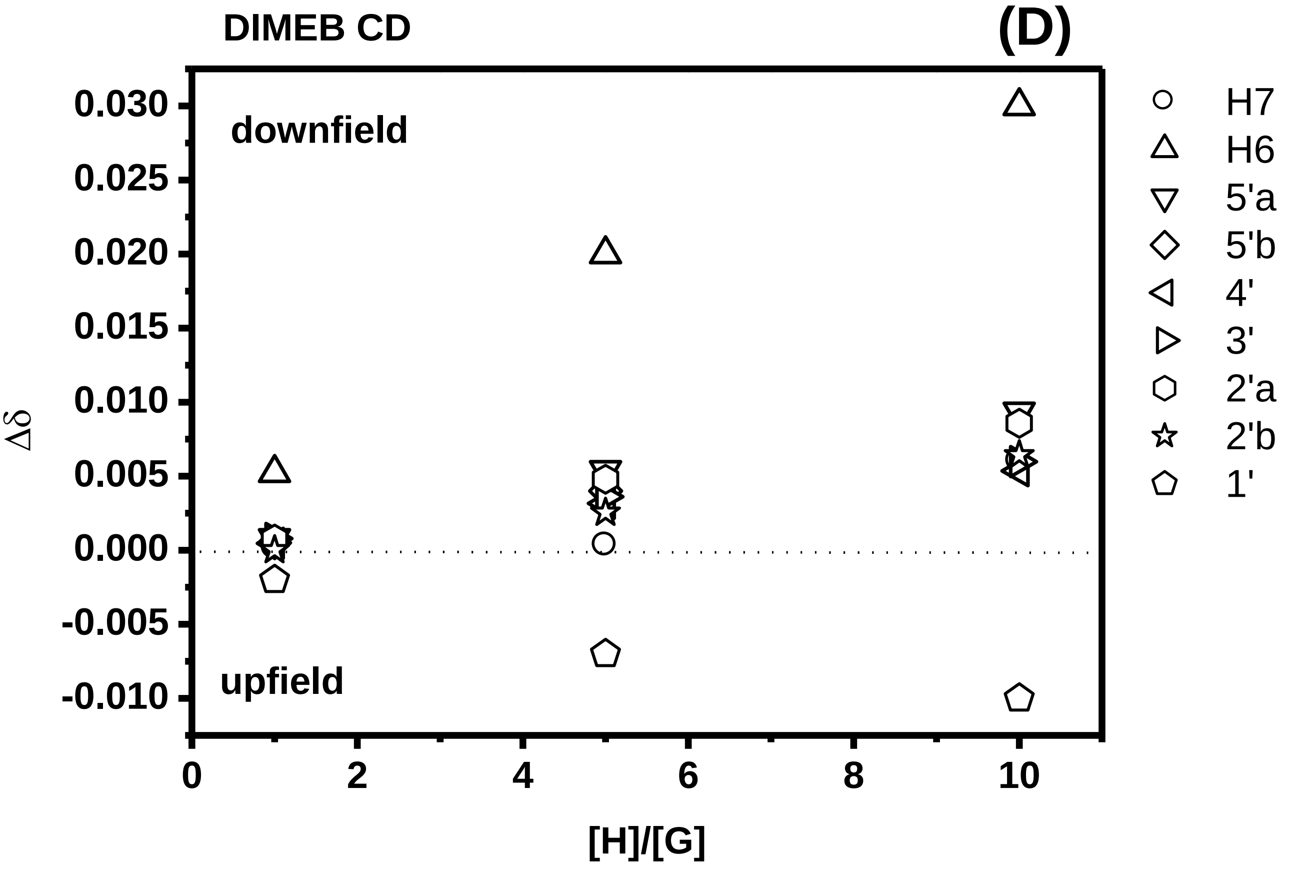
DIMEB CD
(D)
downfield
upfield
Δδ
[H]/[G]
0.030
0.025
0.020
0.015
0.010
0.005
0.000
-0.005
-0.010
0
2
4
6
8
10
H7
H6
5'a
5'b
4'
3'
2'a
2'b
1'

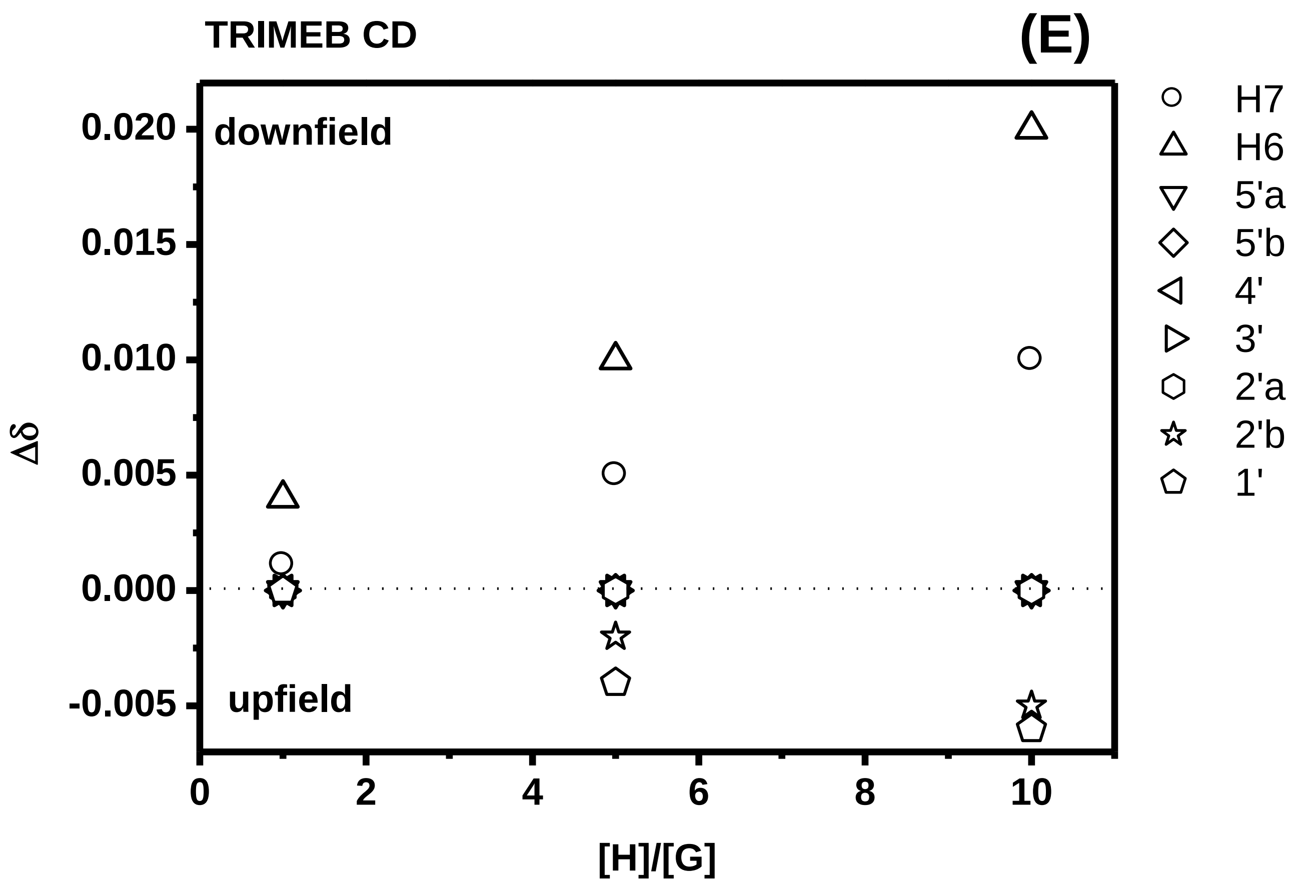

Figure S1: Differences between the observed chemical shifts of S^4TdR protons (see Figure 1 for proton labeling), in presence of the CDs, i.e., $\Delta\delta = \delta_{(complexed\ state)} - \delta_{(free\ state)}$. **(A)** 2-HP-α-CD/$S^4$TdR, **(B)** 2-HP-β-CD/$S^4$TdR, **(C)** 2-HP-γ-CD/$S^4$TdR, **(D)** DIMEB CD/$S^4$TdR, **(E)** TRIMEB CD/$S^4$TdR inclusion complexes. The reported host/guest ratios are 1:1, 1:5 and 1:10.
